# The reimbursement for expensive medicines: stakeholder perspectives on the SMA medicine nusinersen and the Dutch Coverage Lock policy

**DOI:** 10.1186/s12913-022-08690-z

**Published:** 2022-11-04

**Authors:** Féline E. V. Scheijmans, Margot L. Zomers, Sina Fadaei, Marthe R. Onrust, Rieke van der Graaf, Johannes J. M. van Delden, W. Ludo van der Pol, Ghislaine J. M. W. van Thiel

**Affiliations:** 1 Department of Neurology, UMC Utrecht Brain Center, University Medical Center Utrecht, Utrecht University, Utrecht, The Netherlands; 2 University Medical Center Utrecht, Heidelberglaan 100, 3584CX Utrecht, The Netherlands; 3 Department of Public Health, Healthcare Innovation & Evaluation and Medical Humanities, Julius Center for Health Sciences and Primary Care, Utrecht, The Netherlands

**Keywords:** Healthcare policy, Expensive medicines, Rare disease, The Netherlands, Reimbursement policy

## Abstract

**Background:**

The reimbursement for expensive medicines poses a growing challenge to healthcare worldwide. In order to increase its control over the costs of medicines, the Dutch government introduced the Coverage Lock (CL) policy in 2015. The CL postpones decisions regarding reimbursement of expensive medicines until detailed advice on i.e., cost-effectiveness has been given. The CL has been in place for six years, has raised many questions and concerns, but currently, no evaluation is known to the authors. A better understanding of the effects of the CL on all stakeholders involved may contribute to reflections on the CL process and help find ways to improve it. An evaluation of Dutch policy will also be relevant for other countries that aim to optimize reimbursement procedures for expensive treatments. To perform this evaluation, we focused on the CL procedure for the medicine nusinersen. Nusinersen is the first treatment for spinal muscular atrophy (SMA). Following EMA approval in May 2017, it was placed in the CL. The analysis of cost-effectiveness and added therapeutic value resulted in an advice for reimbursement limited to children younger than 9.5 years at the start of treatment; this was implemented from August 2018 onwards.

**Methods:**

Qualitative stakeholder perspective analysis of the CL procedure focusing on nusinersen with 15 stakeholders.

**Results:**

Stakeholders raised key issues of the CL based on their experience with nusinersen: emotional impact of the CL, duration of the CL procedure, appropriateness of the CL procedure for different types of medicines, transparency of the CL, a wish for patient-centred decision-making and the lack of uniformity of access to expensive treatments.

**Discussion:**

Stakeholders supported measures to control healthcare expenses and to ensure reasonable pricing. They considered the delay in access to therapies and lack of procedural transparency to be the main challenges to the CL. Stakeholders also agreed that the interests of patients deserve more attention in the practical implementation of the reimbursement decision. Stakeholders suggested a number of adjustments to improve the CL, such as a faster start with conditional reimbursement programs to ensure access and intensify European collaboration to speed up the assessment of the medicine.

**Supplementary Information:**

The online version contains supplementary material available at 10.1186/s12913-022-08690-z.

## Background

A relatively new challenge for solidarity-based healthcare systems is to incorporate very expensive medicines for rare diseases [1–6]. In the Dutch healthcare system, based on solidarity, citizens pay taxes and mandatory health insurance premiums to finance accessible healthcare for everyone. However, the growth in the number of medicines for rare diseases requires an increasing percentage of the healthcare budget, which risks crowding out other types of healthcare [2, 3, 7–10].

Governments need policy-instruments to guide decisions on which medicines should be reimbursed. The Coverage Lock (CL) is an example of a policy-instrument that was implemented in the Netherlands in 2015 [11]. The purpose of the CL is to evaluate new, expensive medicines, that are used in hospitals (inpatient medicines) before inclusion in the basic health insurance. Price negotiations are also part of the CL. New medicines that exceed a certain budget limit – a budget impact of more than €40,000,000 in total or more than €50,000 per patient resulting in total costs exceeding €10,000,000 – are placed in the CL directly after market authorization by the European Medicine Agency (EMA). In the CL the new medicines undergo an additional scientific assessment and appraisal by the Dutch Healthcare Institute (DHI) based on four criteria: efficacy, cost-effectiveness, necessity and feasibility (financial and practical). This results in an advice to the minister of Health on the reimbursement and pricing. Subsequently, the ministry of Health may decide to start price negotiations with the pharmaceutical company involved [11]. The CL postpones reimbursement, and with that, access to treatment, until these steps have been finalized and a final decision has been made by the Minister for Health. Access to treatment during the CL is sometimes possible, but only by participating in a clinical trial (if available) or a compassionate use program, financed by the manufacturer.

The CL was used in 2017 for the first available treatment, nusinersen, for hereditary proximal spinal muscular atrophy (SMA). SMA is an autosomal recessive neuromuscular disorder caused by the loss-of-function of the survival motor neuron 1 (*SMN1*) gene. With an incidence of 1:6000–1:10,000 new-borns, it is a rare disease but still one of the more common genetic disorders that affects infants and young children. The most common and severe form, SMA type 1, causes respiratory failure in the first years of life, whilst the slightly milder chronic forms, types 2 and 3, cause stalled gross motor development followed by gradual deterioration in muscle strength and severe levels of disability [12–14]. The efficacy of nusinersen was demonstrated by two small studies on the basis of which the EMA granted market access in May 2017 [15, 16]. Nusinersen has a list price of €499,800 in the first year for six injections, followed by €249,900 for three injections per year per patient.

In the Netherlands, the CL procedure for nusinersen lasted 15 months (May 2017-August 2018) and resulted in a reimbursement arrangement for children with SMA who started treatment before the age of 9.5 years, thus excluding older children and adults, in contrast to all neighbouring countries (Germany, Belgium, United Kingdom). The duration of the procedure and the medicine being available through a special arrangement for only a very small group of children caused concerns for parents of other children with SMA and older patients. When the final reimbursement was arranged and an age limit was set, it split families with two children with SMA in half. These cases generated media attention for the CL and gave rise to a public debate (see e.g., [17–19]). The case of nusinersen was a high-profile case and therefore interesting to evaluate the CL. As far as we know, the CL has not been evaluated since its introduction in 2015. Because of the increasing use of the CL due to the growing number of expensive treatments, we decided to evaluate the experiences and perceptions of stakeholders in the CL procedure of nusinersen in the Netherlands. An evaluation of Dutch CL policy is also relevant for other countries that aim to optimize reimbursement procedures for expensive treatments [5, 20].

## Methods

### Identification and inclusion of participants

In order to better understand similarities and differences in the experience and perception of stakeholders regarding the exact same procedure, we screened participants in the CL procedure for nusinersen (FS, WLP, GvT). We used purposive sampling to find parties and persons representing these parties who were specifically involved in the nusinersen dossier and are well known with the CL policy [21]. Parents of patients were contacted through the SMA centre of expertise in Utrecht.

All parties involved in a CL procedure were represented by the selected stakeholders. The identified stakeholders were contacted through email and after obtaining informed consent, an interview was scheduled. All invited participants accepted to participate. Informed consent was validated and reconfirmed orally at the start of the interview and recorded.

### Interviews and data collection

We conducted semi-structured interviews. The interview guide was designed by two authors (MO, SF) and evaluated by two other authors (GT, FS). It consists of questions about the role participants had in the procedure, their experiences, the perceived necessity and strong and weak elements of the procedure (see supplementary file 1 and 2). After carrying out three interviews, we analysed the transcripts to ensure the interview guide fitted the research aim (MO, SF, FS, GT). No adjustments were made. The interviews were conducted by three authors (MO, SF, FS), one of whom (FS) was present at all interviews to ensure continuity. Data collection was stopped when respondents from all stakeholder perspectives had been included. Thematic saturation was reached after interviewing 15 stakeholders. The interviews were transcribed verbatim for further data analysis by one of two authors (MO, SF) or a professional organisation (uitgetypt.nl).

### Data analysis

The first nine transcripts were independently coded by two authors (MO, SF) using Nvivo 12. The code-tree was constructed by inductive coding. Two other authors (MZ, FS) (re-)read the transcripts and adjusted the code-tree. Using the adjusted codes, all interviews were re-coded by one author (FS) and randomly checked by two authors (MZ, GT). Thematic analysis followed after comparison across the interviews and the identification of higher order themes [22]. The stakeholders were divided into three groups representing a patient, clinical and policy perspective. The patient perspective consisted of parents of young SMA patients and a representative from a patient organization. The clinical perspective consisted of caregivers involved in the treatment of SMA patients in the Netherlands. The policy perspective consisted of stakeholders from different organizations, governmental, manufacturer and healthcare insurers point of view. If views within one of the perspectives differs, we make this clear in the result section.

Themes that were common to these three perspectives were identified as the key issues of the CL by two authors (MZ, FS) and discussed with four other authors (JD, WP, GT, RG). After the first analysis we organised an online stakeholder meeting to present the results and verify the themes with the stakeholders.

### Ethical approval

According to Dutch law, interview studies are exempt from review by a Medical Research Ethics Committee (MREC) in case the questions are non-invasive cause no potential harm for participants. The MREC METC Utrecht (currently NedMec) confirmed this in a decision (nr. 20/696) on 11 November 2020.

## Results

We conducted 13 interviews involving 15 respondents between November 2020 and April 2021. Due to COVID-19 restrictions, all interviews were conducted online and lasted 27–75 min. Table 1 presents characteristics of the stakeholders. The stakeholder meeting to verify and confirm the results was attended by six stakeholders. The themes described below were shared and confirmed by the attendees. No new themes were added.

**Table 1:** Stakeholder characteristics

**Patient perspective**
5 interviews: 4 with parents of patients with SMA 1 with a representative of the patient organisation
**Clinical perspective**
3 interviews: 2 with nurses involved in SMA treatment in the SMA Centre of Expertise in Utrecht 1 with a physician involved in SMA treatment in the SMA Centre of Expertise in Utrecht
**Policy perspective**
5 interviews: 3 with representatives of the governmental organisations involved (5 persons, Ministry of Public Health and Sports and the Dutch Healthcare Institute) 1 with representative of the pharmaceutical company involved (Biogen) 1 with a representative of one of the largest health insurance companies (Zilveren Kruis)

Overall, stakeholders acknowledged that healthcare expenses have to be controlled; most participants agreed that the CL might be an effective way of doing this and thus might contribute to affordable care. Further experiences and perceptions from stakeholders can be traced back to six overarching themes described below (for illustrative quotes see Table 2).

**Table 2:** Illustrative quotes from interviews with stakeholders

**Emotional impact of the CL on stakeholders**
Q1: “…For us, that was a very intense period because you just feel powerless, because you know there is something and I cannot buy it at the supermarket; how can I get it, I just want my child to get it. We were really desperate at that time because our child was getting worse.” **(P)** Q2: “Once that decision was made, and our child would miss out, then we actually thought: what are our alternatives? […] So time became more and more urgent and we thought we would emigrate to Belgium. We were actually completely fed up with being in the Netherlands, so we took the necessary steps. We went to see where we could live and what was needed for that, because in Belgium all patients were reimbursed.” **(P)** Q3: “I mean you might be able to formulate a cut-off limit with it, but is this meaningful enough to override the doctor's duty not on improper grounds, yes, everyone's of course, but the doctor still has that duty himself and you probably feel that too. Somehow it hurts that it is not fair. **(C)**
Duration of the CL procedure
Q4: “Look, there was no cure for SMA. It is a progressive disease and if you yourself take 2 years, and—I'll just say—at your convenience, to reach your decision: that is unacceptable. Look, I can imagine if you have a disease of your eyes for example, and there is a drug that makes you better, but in the meantime it doesn’t get worse. That it is recoverable, but with a progressive disease that is irreversible, time is just key.” (**P**)
CL procedure is not appropriate for all types of medicine
Q5: “But what happened with spinraza: because it worked so well, it was assessed at an accelerated pace. Not only in America but also in Europe this was quickly assessed by the registration authorities. The result was that at the time there was a permit, there had been no publication yet. Because normally the process takes 10 months, then you still have time, but we were at 6 months by then. So we had to wait for the publication. […] At such a moment, the health care institute says: sorry, but the file is not complete. So we cannot assess it yet. This gave a month’s delay and was very frustrating.” **(B)** Q6: “You will not see that in rare diseases such as a muscle disease where the muscles are simply damaged and can no longer recover. So, stabilization or slower decline is already a huge advantage. I had to explain that. […] So, in other words, in rare diseases, stabilization, being able to turn over in bed, is a huge gain. Or being able to take three more steps, meaning you can still go to the toilet yourself. That is a huge gain. That is very difficult to explain if you do not know what the disease entails and if you look at the outcome of the data, you think: stabilization is “mwah”. At that point therapeutic value is set at less than the cost. But that is not the case, because it has an enormous therapeutic value for this disease. So, I do think you should look at data with a good eye, and I think that is fairly rigid now.” **(P)**
Transparency of the CL
Q7: “I do not know what happens in the time of the coverage lock. That is speculation for me too.” **(P)** Q8: “But when I ask colleagues, that is what I hear a lot from guys: it’s not always that clear. What I miss in the Coverage Lock is something like: this is what the process looks like, these are the milestones, they are achieved on this day and this day.” **(B)**
A wish for patient-centred decision-making
Q9: “And that mainly concerns, which I find very difficult, that decisions are made about, for example, SMA typing, so type 1 yes, type 2 no, by people who have no idea at all what the typing of SMA means.” **(C)** Q10: “(…) of whom you actually think: they deserve that treatment, but that is not allowed according to the rules. And that is also very difficult to explain to people. So there are, on all sides as you notice, people around the table, people who do not quite, yes, understand the human dimension or the essence of that patient, so to speak.” **(C)** Q11: “That is complicated for the government, which has the formal task of doing the assessment. They should keep doing it, because they are the only ones who can do the assessment objectively.” **(B)**
The lack of uniformity in access to expensive treatments in European countries
Q12: “Then I found it very difficult to see on the internet that there were people in other European countries who were adults, had type 3, who were given an injection for the tenth time. While there were young children in the Netherlands who did not get anything yet.” **(P)** Q13: “I myself became enamored with the German model, where they say: we are putting the patient first, so we are going to start treatment and then we take two years to make a decision. Then we will investigate. So we are not going to wait two years until we make a decision. The patient must not become the victim of our bureaucratic processes. I would find that ideal. Of course, it has drawbacks; I understand that too.” **(P**)
Translating the themes into improvements of the CL in the future
Q14: “I can imagine that you check: is there already a medicine on the market? If there isn’t, then go ahead, then start treatment quickly and negotiate a price. And B if there are drugs on the market, but there will be a new drug that is easier in administration and also cheaper, yes then you have to be quick too, I think.” (**P**) Q15: “Yes, it will help, I think, because the Netherlands is simply too small if, for example, you unite as European countries and then start negotiations with a larger body with pharmaceutical companies. Then you are just much stronger. […] Well, I think you cannot avoid critical assessment, but the question is whether that should only be done from the Netherlands. Why not do the same with a number of countries. That you say: I work together with Germany and Belgium, as is happening now, for example, with gene-therapy in the Benelux.” **(C)**

Legend: *P* Patient representative, *C* Clinician, *B* Stakeholder from policy perspective

### Emotional impact of the CL on stakeholders

Clinicians and patient representatives described their experience with the CL primarily in terms of emotional impact. Feelings of uncertainty, powerlessness and unfairness were mentioned by parents. They experienced the lengthy procedure as nerve-racking and lived between hope and fear (Q1, Table 2). Not being able to influence the procedure contributed to their feeling of powerlessness. Some parents mentioned they had been thinking of emigrating to another country to obtain access to treatment for their (severely) affected children (Q2, Table 2). Parents whose children eventually received reimbursement for the treatment found it difficult to feel happy and relieved. They sympathised with parents and their children who had the same desperate unmet need but who were too old.

Feelings of unfairness were mentioned by parents and clinicians regarding the age limit of 9.5 years in the reimbursement arrangement. In some families, a younger child had access while an older sibling did not. Clinicians and parents experienced this as harsh and difficult to understand.

Clinicians felt frustrated about the age limit in the reimbursement arrangement, which they found to be incomprehensible and highly theoretical. This feeling was further aggravated by the internal conflict of knowing that treatment is available but not being able to prescribe it (Q3, Table 2). Several patient representatives described a similar experience.

Policymakers had the impression that patients or their parents had amplified expectations of the efficacy of the treatment based on examples found on social media. They thought that these high expectations fuelled the frustration of patients or their parents about the delay in access to treatment.

### Duration of the CL procedure

Participants from all perspectives mentioned the duration of the decision-making as one of the key issues of the CL; it takes too long. A time-consuming procedure like the CL was considered to be undesirable in general, but certainly when it involves assessing a treatment for a progressive disease like SMA that affects children (Q4, Table 2). Clinicians mentioned that for a child aged 1.5 years, having to wait almost 1.5 years before reimbursement is arranged, is disproportionately long. Clinicians explained that this experience with nusinersen causes uncertainty among patients and themselves about the duration of future CL procedures for SMA-treatments.

Stakeholders from all perspectives perceived the procedure to be thorough. One governmental policymaker emphasized that acceleration of the procedure in the future should not be at the expense of comprising the thoroughness and precision of the assessment.

### CL procedure is not appropriate for all types of medicines

Policymakers and patient representatives explained that the EMA and the assessors in the CL procedures differ with regard to the level of unpublished data acceptable for efficacy assessment. They mentioned that the EMA accepts unpublished efficacy data for the assessment in the case of orphan medicines. They explained this is done to accelerate authorized market access, as was the case with nusinersen (Q5, Table 2). They also stated that unpublished data have limited and relative weight in the CL; published data are required for the efficacy assessment. To avoid a delay in the CL assessment, participants from all perspectives agree that the CL should also accept unpublished data on efficacy of orphan medicines.

Patient representatives added that the methodology of cost-effectiveness, i.e., Quality Adjusted Life Years (QALYs), is rigid and less suitable for evaluating a medicine like nusinersen because this approach may undervalue effects of nusinersen on the quality of life and inflate the negative cost-effectiveness ratio (Q6, Table 2).

### Transparency of the CL

Some parents felt there was a lack of transparency; they indicated that they did not know exactly what the CL entails nor what happens behind the scenes (Q7, Table 2). A clinician had the same experience: it was not clear who is involved in the CL nor how healthcare provision is ensured during and after the CL. Policymakers from outside the government expressed a lack of clarity about what exactly is expected of the parties involved in the CL and at what stage. They mentioned that there is no overview of what the procedure looks like, the milestones of the CL or the time it takes (Q8, Table 2). As a result, they felt that it is impossible to hold each other accountable for carrying out tasks in the CL.

Stakeholders from all perspectives mentioned that medicine prices, not only nusinersen but other medicines too, are not transparent after the CL. This made them question how effective the CL is in cost management.

Some patient representatives attended the final public CL meeting on nusinersen and perceived this to be important for transparency. Sharing the assessment reports was considered to be an indication of transparency by one clinician. Some participants from the policy perspective, involved in governmental organizations, emphasised that they tried to maintain transparency by updating the patient organisation and families and streamlined information during the procedure.

### A wish for patient-centred decision-making

Patient representatives and clinicians in particular mentioned the lack of the human dimension in decision-making about the CL (Q9, Table 2). Patient representatives experienced that the decision-makers had no idea of the impact of decisions on families and that insufficient attention is paid to the interest of the patient or ethical considerations. This experience was confirmed by clinicians. They emphasised the unworkable practical application of CL decisions and attributed this to the focus on cost control in the CL (Q10, Table 2).

Patient representatives and clinicians expressed a wish for greater involvement of parents, the patient organisation and clinicians in the CL procedure. In the current situation, clinicians feel the decision is imposed on them. Clinicians were asked to provide information about the disease and potential effect of the treatment, but were not involved in the decision-making. Clinicians experienced that they were called to account for the decision that was made, while they in fact had no influence on, and were not responsible for, the final CL decision.

Some policymakers, from governmental organizations, indicated that CL decisions are often made under considerable societal pressure. Furthermore, they felt there was a constant threat that the pharmaceutical company would end the availability of the medicine in a compassionate use program. One of the policymakers, from a governmental organization, mentioned being approached personally by parents and patients, and experienced this as difficult, considering the job of a policymaker is supposed to remain impartial.

Stakeholders from all perspectives appreciated the neutrality, impartiality and independence of the governmental parties currently involved in the CL procedure (Q11, Table 2).

### The lack of uniformity in access to expensive treatments in European countries

Participants from all perspectives mentioned the lack of uniformity between EU member states in accessing expensive treatments as being an important problem. Patient representatives expressed astonishment and horror that social media showed the availability of nusinersen for a broad range of patients (including adults) in other European countries, while young children in the Netherlands did not have access (Q12, Table 2). This astonishment was shared by clinicians. Patient representatives felt that this lack of uniformity was unacceptable.

Patient representatives considered the German policy – allowing treatment to start while further assessment and price negotiations were taking place—as an approach which did not sacrifice the interest of the patient for bureaucratic procedures (Q13, Table 2). Policymakers from governmental organizations suggested that this approach might be attractive from the pharmaceutical company’s point of view. The fact that the medicine has to be taken away from patients if the decision is negative, was seen as a disadvantage of the German policy by patient representatives and policymakers.

Some policymakers mentioned that, compared to other European countries, the Netherlands holds an overall top position with regard to the package of medicines reimbursed, the time required to ensure access to these medicines and their price.

Policymakers had the impression that the authorities of countries with faster access to expensive therapies than the Netherlands have a higher level of trust in the EMA and professionals. They also had the impression that authorities in these countries are more open towards re-assessing the decision after some time on the basis of real-world evidence gathered in, for example, national registers.

### Translating the themes into improvements of the CL in the future

Most of the stakeholders came up with suggestions for future improvements while talking about their CL experiences and perceptions.

Making a timely decision was mentioned often as an important improvement. Patient representatives suggested that timely decisions could be assured by distinguishing treatments for progressive diseases (in which time is key) from diseases that are not or less progressive and to prioritize the first group over the second. Clinicians and patient representatives suggested an alternative trajectory for treatments for (orphan) diseases with no other treatment options. An example of such a trajectory was a conditional reimbursement program (Q14, Table 2). This solution was also proposed by clinicians and policymakers to deal with limited available efficacy data. To take uncertainty around (cost-) effectiveness into account, policymakers proposed a pay-for-performance arrangement.

Stakeholders from all perspectives suggested that EU countries could collaborate (more) in the assessment of the (cost-)effectiveness of expensive medicines and join each other in price negotiations. It was thought this would be more efficient and might strengthen their position (Q15, Table 2).

## Discussion

With this study we aimed to gain insight into the stakeholder perspectives on a Dutch policy measure to control reimbursement for extremely expensive new medicines, known as the Coverage Lock. Overall, stakeholders acknowledged that healthcare expenses have to be controlled; most participants agreed that the CL might be an effective way of doing this and thus might contribute to affordable care. Furthermore, we identified six overarching themes that emerged from the experiences and perceptions of stakeholders of the CL procedure for nusinersen. Our study reveals that even though stakeholders are aware of the necessity to control healthcare expenditure, the CL places strains on stakeholders of all perspectives. Patients and their parents feel powerless; waiting for a (lifesaving) treatment is very difficult. The decision of the CL is imposed on clinicians, who experience an unworkable situation in their clinic. And policymakers from governmental organisations feel societal pressure and are unsure whether treatments, available in a compassionate use program, will remain available. The reimbursement decision on expensive medicines has to be made despite all these tensions in the CL procedure. Above all, clinicians and patient representatives experienced the CL as unfair, especially in comparison to neighbouring countries, because while the procedure is in progress, a (potential) treatment is withheld.

The importance of a fair procedure to come to reimbursement decisions has been described before. An often-proposed ethical framework for such a process is accountability for reasonableness (A4R) (see Table 3) [23–26].

**Table 3:** Accountability for reasonableness framework requirements

*This framework consists of four requirements to come closer to a fair decision-making process about the reimbursement of therapies *[23]*:*
*1. Publicity: meaning the reimbursement decisions must be publicly accessible and ensures clarity about reasons behind a reimbursement decision*
2. *Relevance, also called reasonableness. This condition asks for a reasonable explanation of how the organisation provides “value for money” in meeting the varied health needs of a defined population under reasonable resource constraints. Specifically, an explanation is reasonable if it appeals to reasons and principles that are accepted as relevant*
3. *There should be a mechanism to challenge and dispute the resolution regarding limit-setting decisions, this includes the opportunity for revising decisions in light of further evidence or arguments*
4. *There should be a voluntary or public regulation of the process to ensure that conditions 1 to 3 are met*

When we compare the stakeholder experiences and perceptions to A4R requirements for a fair process, some of the pitfalls of the CL become clear. Most problems expressed by the stakeholders relate to the A4R conditions, publicity (criterium 1) and relevance (criterium 2). Although the final decision in the CL procedure is made public, the CL procedure itself is not always clear and transparent for stakeholders. For some stakeholders it was not transparent to them how the procedure works, what the milestones are or who participates in the process. Governmental organisations tried to inform the stakeholders, but insights derived from other stakeholder perspectives have revealed that this information process was not sufficient. The identification of transparency as one of the key issues of the CL is in line with other literature that indicates the need for transparency about the reimbursement of expensive treatments [4, 24, 27].

With regard to relevance, it could be argued that the CL did not meet this condition in the case of nusinersen. Clinicians and patient representatives in particular were clear that they did not understand the reasoning for an age limit for reimbursement set at 9.5 years. Relevance also appeals to reasons and principles that are accepted as being relevant. For example, patient representatives argued that the CL does not take into account patient relevant outcome measures, which can be considered as relevant in a reimbursement decision. It has been described before, that the criterium relevance is not always met in reimbursement decisions for orphan medicines [28]. Especially the arguments most often used to reason a reimbursement decision, cost-effectiveness, can be difficult to assess in orphan medicines [26, 29, 30].

Stakeholders expressed fewer experiences that can be related to the two other criteria from A4R, revisability (criterium 3) and public regulation (criterium 4). This might be explained by the conditional reimbursement of nusinersen for patients older than 9.5 years that started in 2020; the eventual availability of the medicine makes these two conditions less urgent for stakeholders who are directly involved.

While discussing the strains the CL places on stakeholders and potential improvements of the CL, it is important to realize that solutions to improve the situation of stakeholders might also be found elsewhere. Ultimately, the origin of the problem is the unexplained high price of orphan medicines [9, 31–33]. Pharmaceutical companies should not only clarify pricing of orphan medicines but also share a responsibility to come up with solutions that allows the incorporation of innovative medicines in realistic healthcare budgets [34, 35].

One of the strengths of our study is that we were able to identify and interview the key stakeholders in the CL of nusinersen, thus ensuring valuable insights from people actually involved in the CL rather than hypothetical or theoretical situations. Another strength is that we were able to verify the identified themes with the interviewees. A limitation is the time-interval between the CL procedure of nusinersen and our interviews, which was almost two years. This introduces a risk that stakeholders may not be able to recall the experience in full, or may remember it differently from the way they experienced it at the time of the CL. However, during the conversations with stakeholders, we did notice that details of their experiences came back. We, therefore, asked participants to contact us if anything came up later or if they remembered more after the interview. None of them did; and so we believe we managed to obtain an accurate overview of the experiences of all stakeholders*.*

## Conclusion

In conclusion, stakeholders accept the need for cost-control. Improvements in the CL can, however, be made, especially regarding publicity and transparency. Providing more information about the CL procedure, the timeline with milestones and transparent medicine prices would contribute to a better understanding of future CL procedures. Another important improvement relates to relevance. Efficacy and cost-effectiveness analyses, as instruments in the CL, were perceived as not suitable for all types of treatment, especially not for orphan medicines. A revision of the assessment procedure for orphan medicines is recommended, taking more patient-relevant outcomes into account. Finally, what seems to be of most help to patients and clinicians, is taking timely CL decisions, and ensuring treatment access during the CL procedure.

## Supplementary Information

**Additional file 1:Supplementary file 1.** Interviewguide in Dutch. **Supplementary 2.** Interview guide translated from Dutch to English.

## Data Availability

The datasets (transcripts) generated and analysed during the current study are not publicly available to ensure privacy of the interviewed stakeholders, but are available from the corresponding author on reasonable request.

## Abbreviations

A4R: Accountability for reasonableness
CL: Coverage Lock
EMA: European Medicine Agent
SMA: Spinal muscular atrophy
QALY: Quality adjusted life year
