# Supplementary material for: The reimbursement for expensive medicines: stakeholder perspectives on the SMA medicine nusinersen and the Dutch Coverage Lock policy
Source: BMC Health Serv Res. 2022 Nov 4;22:1320. doi: 10.1186/s12913-022-08690-z (PMC9636634; doi:10.1186/s12913-022-08690-z)
Supplement: Supplementary file 1 — Additional file 1:Supplementary file 1. Interviewguide in Dutch. Supplementary 2. Interview guide translated from Dutch to English. [file 12913_2022_8690_MOESM1_ESM.docx]

**Supplementary file 1: Interviewguide Dutch**

Introductie

Het onderwerp van dit interview is de zogenaamde Pakketsluis: het beleid van de overheid om bij dure, nieuwe medicijnen eerst nader onderzoek te doen en prijs onderhandelingen te voeren voordat ze vergoed worden via de basisverzekering. Het doel van dit interview is inzichtelijk maken wat de ervaringen en meningen zijn van mensen die betrokken zijn bij de Pakketsluis.

Dit interview wordt strikt vertrouwelijk behandeld, en de antwoorden die u geeft zullen alleen anoniem worden gebruikt. Heeft u op dit moment nog vragen? - Start opname -

Heeft u alles begrepen en geeft u expliciet toestemming voor dit interview?

1. Welke ervaring heeft u met de pakketsluis?

a. In welke hoedanigheid bent u betrokken geweest bij de pakketsluis?

b. Welke concrete ervaringen heeft u meegemaakt?

2. Wat vindt u over het algemeen van het Pakketsluis-beleid?

a. Bent u uiteindelijk positief of negatief gestemd?

3. Welke aspecten van de pakketsluis zijn in uw visie goed?

a. Wat zou niet veranderd moeten worden?

4. Welke aspecten zou u verbeterd willen zien?

a. Heeft u concrete suggesties hoe die verbeteringen eruitzien?

5. Denkt u dat er in Nederland een noodzaak is de Pakketsluis of vergelijkbaar beleid?

a. Waarom wel of waarom niet?

6. Hoe vindt u dat de uitwerking van de pakketsluis in de praktijk is?

a. Bereikt de pakketsluis zijn doel?

b. En op welke manier wel of juist niet?

7. Heeft u nog aanvullende vragen of opmerkingen over de pakketsluis?

Hartelijk dank voor uw medewerking.

**Supplementary 2: Interview guide translated from Dutch to English**

Introduction

The subject of this interview is the so-called Coverage Lock: a governmental policy for assessing expensive, new medicines, and negotiating about the price before deciding on whether they should be covered by basic health insurance. The goal of this interview is to gain insight into the experiences and attitudes of stakeholders involved in the Coverage Lock.

This interview is strictly confidential and the answers you give will only be used anonymously.

Do you have any questions? - Start recording –

Did you understand everything and do you give consent for this interview?

1. What is your experience with the coverage lock?
   1. In what capacity were you involved in the coverage lock?
   2. What actual experiences have you had?
2. What do you think of the Coverage Lock policy, in general terms?
   1. Do you have a positive or negative attitude towards it?
3. Which aspects of the coverage lock are good?
   1. What does not need to be changed?
4. Which aspects should be changed?
   1. Do you have specific suggestions for these changes?
5. Do you think there is a need for the coverage lock or a similar policy?
   1. Why? Why not?
6. What do you think about the implementation of the coverage lock in practice?
   1. Does the coverage lock achieve its goals?
   2. How does it? How does it not?
7. Do you have any additional questions or comments about the coverage lock?

Thank you for your contribution.
